# Study on Low-Temperature Emission Performance of Scandate Cathode with Micro-Blade-Type Arrays

**DOI:** 10.3390/ma13010100

**Published:** 2019-12-24

**Authors:** Zhipeng Lu, Shengyi Yin, Zhaochuan Zhang, Feng Ren, Xinping Lv

**Affiliations:** 1 School of Electronic, Electrical and Communication Engineering, University of Chinese Academy of Sciences, Beijing 101407, China; luzhipeng15@mails.ucas.ac.cn (Z.L.); lvxingping18@mails.ucas.ac.cn (X.L.); 2 Key Laboratory of Science and Technology on High Power Microwave Sources and Technologies, Aerospace Information Research Institute, Chinese Academy of Sciences, Beijing 101407, China; zczhang@mail.ie.ac.cn (Z.Z.); renfeng15@mails.ucas.ac.cn (F.R.)

**Keywords:** scandate cathode, micro-blade-type arrays, laser engraving, thermal-field emission, abnormal schottky effect

## Abstract

In order to meet the requirements of high-frequency vacuum electronic devices with small size, high current density, and low working temperature, a kind of porous tungsten scandate cathode with micro-blade-type arrays was developed. The micro-blade-type arrays were fabricated by laser engraving technology. Subsequently, the cathode was prepared by a vacuum copper removal process and impregnated with active substances at high temperature. Experimental results show that the cathode exhibits excellent low-temperature electron emission performance and that the maximum pulse electron emission current density reaches 81.18 A/cm^2^ at 800 °C. The cathode also shows apparent combined thermal-field emission characteristics. Further analysis shows that a high electric field strength plays an important role in the electron emission of the scandate cathode. By virtue of the electric field enhancement effect formed by the fabricated micro-blade-type arrays on the cathode surface, the prepared cathode achieves high electron emission capacity.

## 1. Introduction

After development for more than a century, vacuum electronic devices (VEDs) have played a key role in many fields, including military, aerospace, communications, scientific research, and other fields. At the heart of vacuum electronic devices are cathodes, including pure metal, oxide, barium tungsten, M-type, and scandate cathodes. The research and preparation technologies and emission properties of cathodes have been continuously improved [1,2,3,4].

With the rapid development of high-frequency VEDs in recent years [5,6], cathodes are required to provide higher emission current density with smaller sizes and lower operating temperatures [7]. In 2015, the US Defense Advanced Research Projects Agency (DARPA) issued the Innovative Vacuum Electronics Science and Technology (INVEST) program [8]. In the program plan, the following performance criteria for cathodes were mentioned: Low temperature (<800 °C), high current density (>20 A/cm^2^), and long life (>10,000 h). This is consistent with the requirements of high-frequency VED electron sources. 

In this context, the traditional M-type cathodes cannot meet the requirements of these devices. Impregnated scandate cathodes are considered as the most promising type of cathode, due to them having the highest electron emission capacity of all kinds of thermionic cathodes. Many scholars have devoted a lot of energy to the research of scandate cathodes; for example, preparation of the scandia-doped tungsten matrix [9,10,11,12,13], fabrication of the active salt [14,15], and top-layer scandate cathodes [16,17,18]. These studies have further improved the properties of scandate cathodes. However, the operating temperatures of these cathodes usually reach 950 °C or higher. Such high temperatures bring potential risks for high-frequency and small-size vacuum devices.

Spindt microtip cathodes and other types of cold-field emission cathodes have a huge advantage in terms of operating temperature [19,20,21,22,23], usually operating at room temperature. However, there are still some prominent problems in these cathodes, such as a complex preparation process, high frequency of discharging and arcing, lack of reliability, and relatively low current density.

In order to obtain high electron emission performance at a comparatively low temperature (below 800 °C), we considered that the advantages of scandate thermionic dispenser cathodes may be effectively combined with those of microtip array cathodes and, consequently, proposed a type of impregnated scandate thermionic cathode with micro-blade-type arrays on its surface. To realize this idea, studies were carried out in this research, including the fabrication of micro-blade-type arrays on the surface of a porous tungsten substrate, preparation of the scandate cathode, characterization of cathode morphology, emission performance testing, and cathode performance analysis. The experiment achieved encouraging results, providing proof that our idea is completely feasible.

## 2. Experiment

### 2.1. Fabrication of Micro-Blade-Type Arrays on Porous Tungsten Matrix Surface 

The material used in this experiment was porous tungsten sponge impregnated with copper. The porosity of porous tungsten matrix was 25–26%.

The main equipment used in the fabrication of the micro-blade-type was a silicon wafer dicing machine with a Nd:YAG fiber laser as the melting source and a mobile worktable. The laser wavelength was 1064 nm, the power was 15.69 W, and the frequency was 40 kHz.

**Figure 1:**
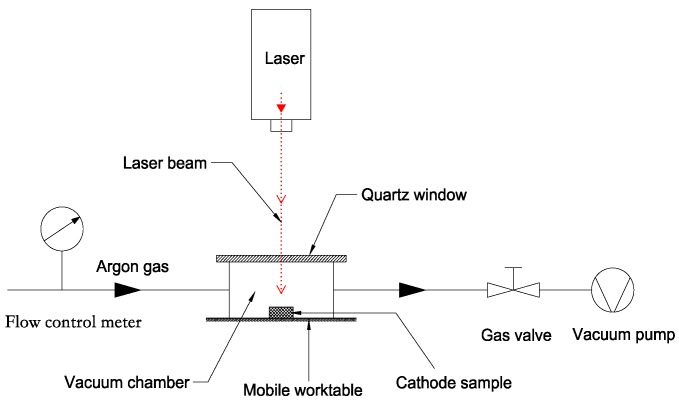
Schematic diagram of laser engraving process.

To avoid the oxidation and contamination of the cathode samples caused by high temperature during laser engraving, a small vacuum chamber was designed, as shown in Figure 1. The top of the vacuum chamber was sealed with a rubber ring and quartz glass, and the chamber was connected with a vacuum pump. The laser entered the chamber through the quartz window.

The manufacturing process was as follows: The tungsten-copper material was processed into a Ø 1 mm flat cylinder, and the emission surface was polished by an automatic polishing system. Subsequently, the sample was placed into the designed vacuum chamber, which had been pumped to 10^−1^ Pa by the vacuum pump. The flow of Ar gas was controlled by a flow control meter, and the trajectory of the moving laser was set and realized by a computer to complete the process of laser engraving. The etching step was set to 40 µm. Then, the tungsten–copper sample with the micro-blade-type arrays was heated to 1600 °C in a high-frequency vacuum induction furnace to remove copper, and the porous tungsten body with micro-blade-type arrays was obtained.

### 2.2. Cathode Preparation

To study the influence of the micro-blade-type array on the performance of the scandate cathode, cathode samples were divided into two groups. In the first group, micro-blade-type arrays were fabricated on the surface of the cathodes, as described in the previous section. The surface of the cathodes in the other group was kept planar. 

In the next step, all samples were impregnated with high active salt containing scandium oxide. The main composition of the salt was BaO–SrO–Sc_2_O_3_–CaO–Al_2_O_3_–ZrO_2_. This kind of salt is based on the previous research of our group in [14]. The impregnation condition was 1800 °C for 2 min.

After impregnation, the residual salt on the cathode surface was removed by an EDTA chemical cleaning method combined with ultrasonic-assisted cleaning. Afterwards, the cathode was reduced in a hydrogen furnace again, where the specification was 1100 °C for 30 min.

### 2.3. Characterization of Cathode Morphology and Performance Test

To characterize the morphology of the fabricated micro-blade-type arrays, a JSM-6510 scanning electron microscope (JEOL Ltd, Akishima, Tokyo, Japan) (SEM) was used to analyze the surface and profile of the cathode. The cathode samples were fabricated with micro-blade-type arrays, and the copper filling the tungsten pores was removed. 

The cathode electron emission test was carried out in a planar diode system with a water-cooled tungsten anode placed in a UHV chamber. The distance between the cathode and anode was about 0.1 mm. The vacuum of the test chamber was pumped to 1.0 × 10^−5^ Pa by an ion pump. A CT-30 power supply (CSIC Ltd, Yangzhou, China) was adopted for the cathode emission performance test. The cathode temperature was non-contact measured by a well-calibrated infrared thermometer. Before the test, the cathodes were all activated at 1150 °C for 60 min, in order to de-gas the anode. After activation, the cathode pulse emission performances were tested at a temperature range of 550–900 °C. The testing voltage range was set to 50–3000 V, the pulse width was 5 µs, the frequency was 20 Hz, and the I/U characteristic data were collected automatically by computer.

## 3. Results

### 3.1. Morphology of Porous Tungsten Micro-Blade-Type Arrays

**Figure 2:**
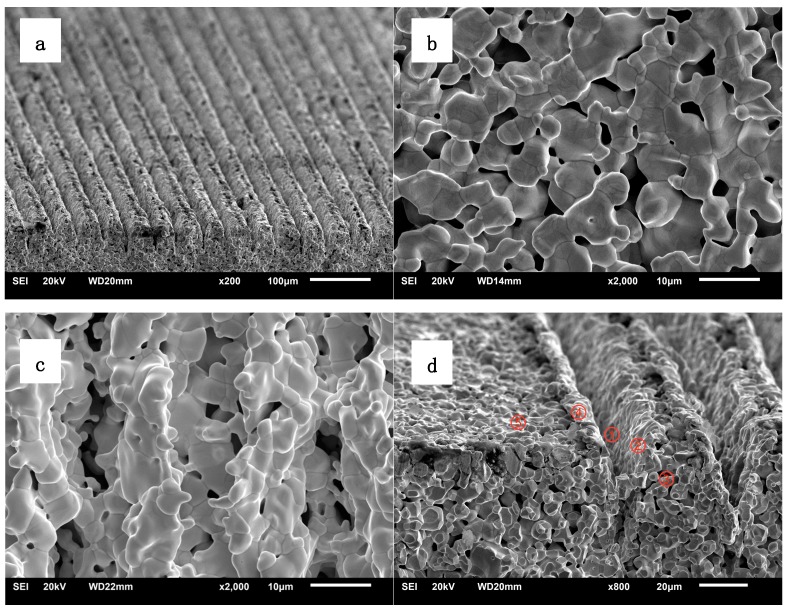
SEM images of the porous tungsten matrix surface: (**a**) Matrix surface with micro-blade-type arrays at 200 times; (**b**) Planar matrix surface at 2000 times; (**c**) Matrix surface with micro-blade-type arrays at 2000 times; (**d**) Matrix surface with micro-blade-type arrays at 800 times.

Figure 2 shows SEM images of the prepared cathode samples taken by the scanning electron microscope at different views and magnifications. The samples shown were not impregnated with active salts. Figure 2a is an overall image of the cathode surface with micro-blade-type arrays at 200 times magnification. From the image, it can be clearly seen that a regular arrangement of micro-blade-type arrays was obtained after laser engraving. According to the scale in the image, we measured the spacing between the micro-blade-type arrays to be about 40 µm, which was consistent with the designed laser engraving parameters. 

Figure 2b,c shows SEM photographs of the pristine planar and micro-blade-type array cathodes, respectively, at 2000 times magnification. Compared with Figure 2b, it is clear that micro-blade-type arrays were formed by laser engraving. Figure 2c shows a more specific morphology of the micro-blade-type arrays, corresponding to the array in Figure 2a. Figure 2d shows the micro-blade-type arrays’ cathode morphology from a cross-sectional view at 800 times magnification, from which it can be seen that the micro-blade-type array was U-shaped and that the profile on both sides was composed of 5–8 tungsten particles (in height) with relatively little porosity. Considering that the size of a single tungsten particle is about 3–5 µm, the estimated tip height is 20–40 µm. From Figure 2c,d, it can be observed that the apex tip of each array was composed of one or two smaller tungsten particles and that the middle region of the micro-blades was composed of porous tungsten material with the same porosity as the matrix.

Based on the above analysis and the characteristics of thermionic dispenser cathodes, it can be concluded that the porous tungsten micro-blade-type arrays obtained by laser engraving technology have the following important characteristics: There are a large number of pores between the tungsten particles which make up the micro-blade-type arrays, especially in the middle of the micro-blades. From the tungsten sponge matrix to the micro-blade-type arrays, the pores remain connected, which provides a diffusion channel for the cathode active substances impregnated in the pore of the tungsten sponge matrix. Furthermore, the U-shaped grooves (10 µm in width and 5 µm in depth) between the two sides of the micro-blade provide favorable conditions for the storage of active substances. 

It can be imagined that, in the cathode activation process, the active material in the U-shaped groove and the pores between tungsten particles, containing Ba and Sc atoms, can easily diffuse from the cathode matrix to the emission surface, reaching the top of the micro-blades and then forming a low-work-function active layer on the surface, which provides the material basis for the cathode to achieve sustained and stable emission.

### 3.2. Results of the Electron Emission Measurements

**Figure 3:**
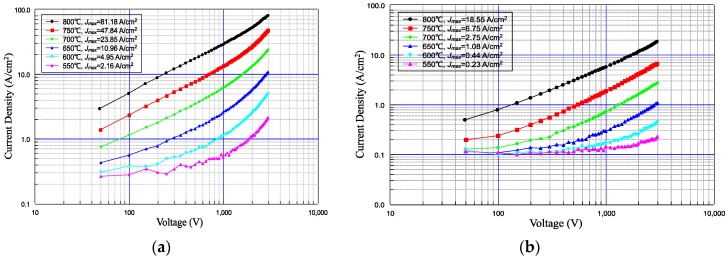
I-V characteristics of the cathodes: (**a**) Scandate cathode with micro-blade-type arrays; (**b**) Planar scandate cathode.

Pulse electron emission properties of the micro-blade-type array cathode scandate and planar cathode samples were tested at 550, 600, 650, 700, 750, and 800 °C. The measured Current-Voltage (I-V) data were processed and plotted to obtain the double logarithmic voltage–current characteristic curves of cathode emission. The results for the two cathodes are presented in Figure 3a,b, respectively.

Comparing Figure 3a,b, it is clear that the cathode with micro-blade-type arrays on the surface had great advantages in terms of electron emission performance. The maximum emission current density of the impregnated scandate cathode with micro-blade-type arrays was 81.18 A/cm^2^ at a temperature of 800 °C, while that of the planar scandate cathode was only 18.55 A/cm^2^ under the same conditions. The current density of the micro-blade-type array cathode was 4.37 times higher than that of the planar cathode. The maximum emission current density of the micro-blade-type array cathode was 4.95 A/cm^2^ at 650 °C, while that of the planar cathode was only 0.44 A/cm^2^, which was less than a tenth of the former. It can also be seen that, when the temperature decreased from 800 to 650 °C, the influence of the micro-blade-type arrays on the emission performance became increasingly obvious, dominating 91% of the cathode emission completely at 650 °C. The emission density of the prepared cathode also exceeded the previously mentioned INVEST specifications for cathodes. 

It is worth noting that for conventional thermionic cathodes, the current density corresponding to the inflection point of the transition from the space charge limited region to the temperature limited region in the I/U characteristic curve of a cathode is usually used as the index to characterize the emission performance of cathode. However, in this experiment, the trend of the voltage–current characteristic curve of the micro-blade-type array cathode at low temperature differed from that of conventional impregnated dispenser cathodes and coated dispenser cathodes. As shown in Figure 3a, the slope of the I/U characteristic curve increases gradually with an increase of cathode voltage, so there was no space charge saturation phenomenon, as with usual thermionic cathodes. Therefore, the above method of characterizing the emission current density of the cathode was not applicable to the fabricated micro-blade-type array cathode in our experiment. For the convenience of comparison, the electron emission current density corresponding to the maximum voltage value of 3000 V of the test system was adopted to characterize the electron emission capability of the prepared cathodes in this paper.

## 4. Discussion and Analysis

### 4.1. Formation of Micro-Blade-Type Arrays

The micro-blade-type array cathode prepared in this paper was a thermionic dispenser cathode. For this kind of cathode, the electron emission and maintenance mainly depend on the active substances stored in the pores between the tungsten particles. The Ba and Sc atoms in these active substances diffuse to the cathode surface by means of the edges of the pores, providing a continuous supplement for the cathode electron emission. If there are no pores, the cathode emission will end in a very short time. Therefore, maximizing the preservation of tungsten sponge pores and forming new pores has become a key technology in the preparation of new types of micro-blade-type array cathodes.

In laser engraving technologies, a suitable laser wavelength, a small focus spot, and a controllable spot trajectory are the basic conditions for realizing laser fabrication. In this experiment, an Nd:YAG fiber laser with 1064 nm wavelength and a mechanical moving part satisfied the above conditions well. When the laser irradiates on the surface of the material, the laser energy is absorbed by the local area of the irradiated material, which heats rapidly within a certain scope of depth. With a rapid heating and cooling process, a series of physical and chemical processes—such as the breaking of chemical bonds between atoms, phase transformation, melting, and gasification—will occur in the material.

It was found that when a porous tungsten matrix without copper was adopted directly for laser engraving, the tungsten particles rapidly absorbed the laser energy and became melted or superheated under the irradiation, which resulting in filling of the original pores in the tungsten matrix, ultimately making it difficult to preserve the pores and form the required micro-blades. Based on this fact, we attempted to use a W–Cu matrix impregnated with copper in the pores between the tungsten particles for laser engraving. The technique allowed us to successfully retain the pores when engraving.

Using the results in Figure 2, the process of laser engraving the tungsten copper matrix to form surface micro-blade-type arrays was analyzed.

As shown in Figure 2d, the laser irradiation area was divided into three regions: 1, 2, and 3, corresponding to the central, sub-central, and edge regions of laser reaction, respectively. The region marked 4 is the surface of untreated tungsten sponge matrix (emission surface). When the focused laser beam irradiated the material surface, the tungsten–copper, labelled as 1, located in the most central region of the laser’s focus rapidly absorbed the laser energy, gasified, expanded, and dug out on both sides, forming the material evaporation region. Simultaneously, a gas jet drove the regional molten tungsten–copper material in region 2 to flow out and form the micro-blades, labelled as 5, after cooling and solidification. Region 3 of the tungsten sponge matrix was barely influenced by the laser heating and showed little change in morphology, as it was far away from the central irradiation area. Finally, the surface micro-blade-type array morphology shown in Figure 2 was formed. It should be pointed out that, in the above process, the laser affected the tungsten particles, and copper filled in their pores simultaneously. Although the melting points of tungsten and copper are quite different, due to the high energy density of the laser, the tungsten and copper did not separate completely during laser engraving. The two materials have similar physical and chemical phenomena: Melting, gasifying, and spraying out and cooling to form micro-sharp arrays simultaneously. According to the above process, it is not difficult to conclude that during the formation of micro-blades, the micro-blades, labelled as 5, remained continuous with the matrix 2 and 3. After the fabrication of the micro-blade-type arrays, a high-frequency copper removal process was adopted to remove the copper stored in the pores between the tungsten particles in the micro-blade-type arrays, thus forming pores connecting the cathode matrix to the micro-blades, as shown in Figure 2b. This provided the conditions for the impregnation, storage, and diffusion of active substances in the processes of high temperature activation and electron emission of the cathode.

### 4.2. Field Enhancement Effect Micro-Blade-Type Arrays

**Figure 4:**
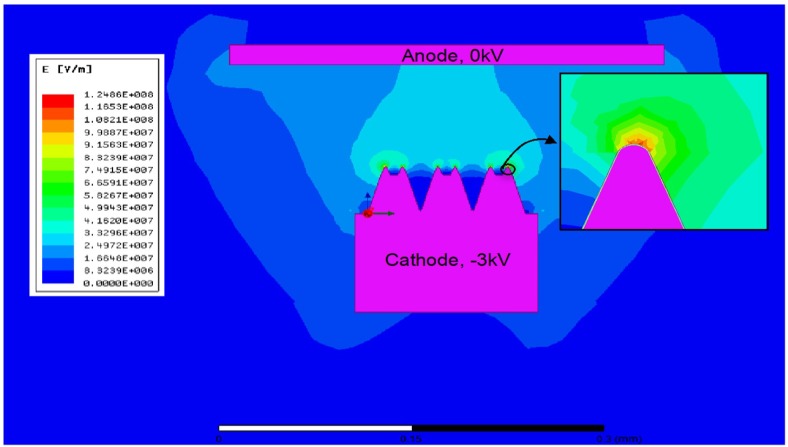
Simulation result of electric emission at the cathode surface.

In order to analyze the effect of the micro-blade-type arrays on cathode surface electric field intensity, a 2D model of the cathode surface micro-blades morphology was established, using the Maxwell 12 software, to simulate the electric field intensity distribution on the cathode surface after voltage was applied between the cathode and anode. The results are shown in Figure 4.

In the model, the size of cathode surface morphology was approximately measured by the ImageJ software [24] from Figure 2 and drawn according to the actual size. The distance between the cathode and anode was 0.1 mm. The anode was grounded, and the cathode voltage was −3 kV. Figure 4 shows that the maximum electric field intensity at the tip of the cathode was more than 1.2 × 10^8^ V/m. Compared with the planar cathode under the same conditions, whose surface electric field intensity was only 3 × 10^7^ V/m, it is quite clear that the maximum electric field enhancement factor generated by prepared micro-blade-type arrays was more than four times. Considering that a cathode’s expansion leads to a decrease of the distance between cathode and anode when heating the cathode, as well as the cathode matrix being composed of tungsten particles with a particle size of 3–5 µm with irregular surface shapes, the surface electric field of cathode may be higher than the simulated results.

In conventional cold-field emission cathodes, the threshold field intensity for producing field electron emission is usually 10^8^–10^9^ V/m [25]. According to the above simulation results, the electric field intensity at the micro-blades of the prepared cathode surface is close to the magnitude of cold-field electron emission. In this view, it is not difficult to understand that the prepared micro-blade-type array cathode shows obvious field emission characteristics.

It should be noted that the electric field of a microtip will decrease in regularly ordered tip arrays, due to shielding effects [26,27]. It can be observed that the highest simulated electric field intensity of the middle micro-blade in Figure 4 was nearly 9 × 10^7^ V/m, which was indeed smaller than that of the tips on both sides. However, the electric field enhancement factor still reached nearly three times. Simultaneously, the reduction of electric field intensity at the middle region of micro-blade-type arrays is greater, which will lead to a reduction in effective emission area. However, there was no reduction in the overall emission current of the prepared cathode, which further reflects the important role played by the electric field enhancement.

### 4.3. Emission Characteristics of Scandate Cathode with Micro-Blade-Type Arrays

Comparing Figure 3a with Figure 3b, we can see that the low-temperature I-V characteristic curves of the planar scandate cathode and micro-blade-type array scandate cathode both presented an “upturned" phenomenon with the increase of voltage. Moreover, this phenomenon was particularly obvious for the micro-blade-type array scandate cathode, which had a more significant increase in emission current density. In this regard, the emission characteristics of the micro-blade-type array scandate cathode were further analyzed. 

The log–log curves at different temperatures in Figure 3 were divided into low field and high field for linear fitting, with the voltage of 1500 V as the boundary. The obtained slopes of the curves are shown in Table 1.

Further analysis of Figure 3a and Table 1 shows that for all the I-V characteristic curves at different temperatures, as the temperature decreases, the slope of the curve at low field also decreases; whereas the slope at high field gradually increases. For a I-V curve at a certain temperature, the slope of the curve increases with increasing electric field, which is inconsistent with the Child–Langmuir law. According to the Child–Langmuir law, when a thermionic cathode is located in the space charge limited region, its log–log plot of voltage and emission current density is linear, where the theoretical slope of the curve is 1.5 [28]. As the accelerating voltage increases, the slope decreases when cathode emission transitions from the space charge limited region to the temperature limited region. For most practical cathodes, the actual slope will be less than 1.5 due to the non-uniform emission of the cathode, but the I-V characteristics generally follow the above rules. However, an opposite phenomenon appeared in this case. From the data in Table 1, we can see that the slope of curve even exceeded the theoretical value of 1.5, which further proves that the field enhancement effect caused by surface micro-blade-type arrays plays an important role in the emission of the prepared cathode. When the temperature and thermionic emission decreased, along with electric field intensity strengthening, the electron emission resulting from field enhancement took up a larger proportion, resulting in the observed increase of the I-V curve. Therefore, there is sufficient reason to believe that the electron emission behavior of the prepared micro-blade-type array scandate cathode is a kind of joint thermal and field emission.

From this point of view, it is easy to understand that for scandate cathodes, the fabrication of micro-blade-type arrays on the surface will lead to a significant increase in electron emission current density.

### 4.4. Analysis of High Emission Characteristics of Scandate Cathode with Micro-Blade-Type Arrays

A large number of studies have shown that, compared with barium–tungsten or M-type cathodes, scandium-containing cathodes present an obvious abnormal Schottky effect; that is, with increasing voltage between the cathode and anode, there is no saturation of the cathode emission current. There have been various explanations for this phenomenon. One of the views is that this effect is caused by the uneven work function at the surface of the scandium-containing cathodes. When the cathode is in the transition zone, some areas of the cathode surface enter the temperature limited zone, but other parts remain in the space charge limited zone [11,29]. From the perspective of a semiconductor model, another viewpoint is that the increase of emission is due to a decrease of the work function in the semiconductor layer caused by external field penetration. It has been considered that the emission of scandate cathodes is due to a combined thermal-field emission, where the electric field plays an important role in the cathode emission [30,31,32].

**Figure 5:**
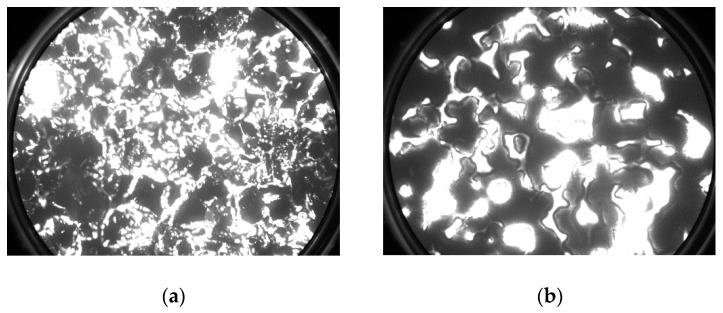
TEEM images of dispenser cathodes (field of view: 100 µm): (**a**) scandate cathode, 660 °C; and (**b**) M-type cathode, 680 °C.

In the previous research by our group on scandate cathodes, we used a deep ultraviolet laser emission/thermal emission electron microscope (DUV-PEEM/TEEM) to analyze the emission morphologies of impregnated scandate and M-type cathodes and found that the emission morphologies of the two types of cathodes are quite different. Figure 5a shows the micro-area thermionic electron emission image of a cathode impregnated with scandium-containing salt at 660 °C. In contrast, Figure 5b is the same kind of image of M-type cathode at 680 °C [33]. The vision field of view of the two images is 100 µm, and the acquisition conditions of the images are the same (same detection voltage of 900 V and exposure time of 2000 ms). As can be seen in Figure 5b, for the M-type cathode, the electron emission region presented a flaky distribution. As for the scandate cathode impregnated with high-emission salt shown in Figure 5a, its electron emission consisted of a large number of dense emission points, which were mainly concentrated at the edges of tungsten particles. We consider the distribution characteristics to be mainly responsible for the abnormal Schottky effect of scandate cathodes.

It can be imagined that these emission points and the edges of particles are exactly the places where the electric field intensity is higher. This indicates that enhancement of the surface electric field intensity plays an important role in promoting the electron emission of scandate cathodes. In [34] and [35], the researchers used a high-resolution scanning electron microscope to analyze the surface of a scandate cathode and found that a large number of nanoparticles were distributed on the surfaces of the tungsten particles. These nanoparticles were densely distributed on the steps and edges of the tungsten particles. Although the compositions of these nanoparticles have not yet been determined, their distribution characteristics were similar to the distribution of electron emission points shown in Figure 5a. As described in [35], the distribution area of nanoparticles in the region will induce a field enhancement effect, which leads to an increase of electron emission current of the scandate cathode. In this respect, compared with M-type cathodes, the field enhancement effect plays a more important role in the electron emission of scandate cathodes. The above findings support the idea that the emission of scandate cathodes is a kind of combined thermal-field emission. In our experiment, the micro-blade-type arrays were fabricated on the surface of the scandate cathode to further improve the field intensity, which is helpful in taking advantage of the properties of the scandate cathode, thus greatly promoting its emission capacity. 

The characteristics of the prepared cathode were similar to those of the thermal Schottky cathode to some extent. In 1969, Lyn Swanson first proposed the Zr/W thermal-field cathode, which has been studied by several scholars [36,37]. Heating of the cathode removed the absorbed contaminants and smoothed out its surface roughness; thus, this kind of cathode could operate in a poorer vacuum and provide stable field electron emission. Simultaneously, high temperatures ranging from 1300 to 1800 K enhanced thermal electron emission. The zirconium and oxygen adsorbed onto the cathode surface also reduced the work function; thus, the cathode emission current was greatly increased. Lida, S and Nagatomi, T studied a type of Sc-O/W(100) emitter used as Schottky emitter [38]. Their research indicated that Sc–O complexes have a lower work function and self-recovery function against residual gas. The phenomenon mentioned above is similar to that observed for the novel cathodes prepared in this paper.

For the micro-blade-type array cathodes, a lower work function layer of Ba–Sc–O was formed in the process of activation and diffusion of active substance [30]. This layer was considered to be sensitive to the field strength. With the combined effects of increasing temperature and high electric field strength formed by the micro-blade-type array, the prepared cathode achieved high current density emission.

To further understand the effect of electric field enhancement of the micro-blades on the emission performance, it is necessary to carry out more in-depth analyses.

For a thermionic emission cathode, its electron emission obeys the Richardson emission equation:

(1) $$J_{0}=A_{0}T^{2}e^{-\frac{\phi }{kT}}$$

where *J*_0_ is the zero-field emission current density, *A*_0_ is an emission constant, *T* is the temperature of the cathode, *K* is the Boltzmann constant, and Ø is the work function of the cathode.

When the applied electric field of the cathode increases to an accelerating field, the surface potential barrier decreases, due to the Schottky effect. Considering this condition, the emission current density follows, as:

(2) $$J=J_{0}e^{\frac{4.4\sqrt{E}}{T}}$$

where *E* refers to the applied field intensity at the cathode.

As mentioned above, many scholars have used the semiconductor model to analyze the emission characteristics of scandate cathodes [31,32]. The model was also adopted here to take the effect of an applied electric field on the emission characteristics of scandate cathodes into consideration. Wright and Wood first used the semiconductor model to explain the emission behaviors of cathodes [39]. The model considers that the penetration of an applied electric field causes a layer of space charge in the low concentration of conducting electrons in the semiconductor layer at the cathode surface, which results in tilting of the semiconductor energy level and reduction of the surface work function. The change of work function *δ_χ_* can be expressed in

(3) $$\delta_{\chi }=-2kTsinh^{-1}\frac{E}{4{\left(2K\pi n_{0}kT\right)}^{1/2}}$$

where *K* is the dielectric constant of the semiconductor layer on the cathode surface and *n*_0_ is the concentration of free electrons in the conduction band of the semiconductor layer.

Furthermore, considering both the reduction of work function caused by the above semiconductor model (3) and Schottky effect under an accelerating field (2), the emission current density is determined as:

(4) $$J=J_{0}e^{\frac{4.4\sqrt{E}}{T}}e^{\frac{\delta_{\chi }}{kT}}.$$

According to (4), the emission current density can be easily calculated under a given electric field intensity. Referring to Figure 4, we calculated the current emission density at the tip. Considering that the electric field intensity of the planar scandate cathode under the same conditions was 3 × 10^5^ V/cm and assuming that the field enhancement factor at the tip is 2.0, the calculated local current density at the tip of the micro-blade-type array scandate cathode was 10.8 times that of the planar cathode. If the field enhancement factor at the tip is considered to be 4.0, the local current density will increase by 177 times (the values of *n*_0_ and *K* refer to [39], where *n_0_* = 10^14^ per cm^3^, *K* = 10, and *T* = 1000 K). It can be seen that the current density increases almost exponentially with an increase of electric field intensity, within the magnitude range of electric field intensity in the experiment. Although it is difficult to quantitatively calculate the overall current density of the cathode, the above results still indicate that the application of an electric field at the cathode surface is of great importance to the electronic emission of a scandate cathode. It is also not difficult to understand that the emission level of the scandate cathode was greatly improved after the micro-blade-type arrays were fabricated on the cathode surface.

## 5. Conclusions

From our study of a new type of scandate cathode with micro-blade-type arrays and its performance in this paper, our conclusions are as follows:

1. An arrangement of micro-blade-type arrays was fabricated well by laser engraving technologies. The micro-blade-type arrays had a height of 20–40 µm and were separated with a spacing of 40 µm. Most of the pores in the tungsten sponge were preserved during the process, which is of importance to the prepared cathode. Simulation results using the Maxwell 2D software showed that the highest electric intensity at the micro-blades was enhanced by four times and reached 1.2 × 10^8^ V/m.
2. The prepared cathode exhibited excellent electron emission levels at low temperatures. According to an analysis of the characteristics of the prepared cathode and TEEM images of a scandate cathode, it was considered that the emission type of scandate cathodes is a kind of joint thermal-field emission, similar to a thermal Schottky cathode. A lower work function layer of Ba–Sc–O was formed and retained by heating the cathode, which was in favor of field emission. With the combined effect of heating in a certain range of temperatures and the high electric field intensity generated by the fabricated micro-blades, the electron emission of the prepared cathode was greatly improved.
3. The semiconductor model was introduced to specifically analyze the influence of electric strength on electron emission. The calculated result indicates that emission density increased greatly with electric field enhancement. The analysis supports the experimental results, where the prepared scandate cathode with micro-blade-type arrays obtained excellent electron emission performance.
4. With the excellent emission performance at relatively low temperature, the proposed cathode has a certain advantage in high-frequency and terahertz VED applications. On the other hand, the fabrication of micro-blade-type arrays may cause the problem of emission uniformity. Work concerning the evaluation and application of the cathode will be carried out in the next step of our research.

## Figures and Tables

**Table 1:** Slope of I-V curves of the micro-blade-type array scandate cathode.

Slope	800 °C	750 °C	700 °C	650 °C	600 °C	550 °C
Slope at low field	0.75	0.77	0.76	0.69	0.55	0.34
Slope at high field	1.03	1.34	1.37	1.50	1.57	1.48
